# Eye blinking in an avian species is associated with gaze shifts

**DOI:** 10.1038/srep32471

**Published:** 2016-08-30

**Authors:** Jessica L. Yorzinski

**Affiliations:** 1 Department of Wildlife and Fisheries Sciences, Texas A&M University, College Station, TX 77843-2258, USA

## Abstract

Even when animals are actively monitoring their environment, they lose access to visual information whenever they blink. They can strategically time their blinks to minimize information loss and improve visual functioning but we have little understanding of how this process operates in birds. This study therefore examined blinking in freely-moving peacocks (*Pavo cristatus*) to determine the relationship between their blinks, gaze shifts, and context. Peacocks wearing a telemetric eye-tracker were exposed to a taxidermy predator (*Vulpes vulpes*) and their blinks and gaze shifts were recorded. Peacocks blinked during the majority of their gaze shifts, especially when gaze shifts were large, thereby timing their blinks to coincide with periods when visual information is already suppressed. They inhibited their blinks the most when they exhibited high rates of gaze shifts and were thus highly alert. Alternative hypotheses explaining the link between blinks and gaze shifts are discussed.

Animals continually monitor their environment for potential threats and opportunities^1^. Despite this active vigilance, they lose access to all visual information every time they blink. Given that blinks can last more than 300 ms and occur 29 times per minute^2^, eyes may be shut during 15% of the time that animals are being vigilant. Blinks are necessary to keep eyes moist and protect against foreign debris^3^,^4^ but they clearly limit the amount of visual information received.

Given this cost associated with blinking, blinking can be strategically timed to minimize information loss^5^. Blinks often occur during gaze shifts, during which time visual information is already being suppressed as eyes shift between targets^6^. They are more likely during large versus small gaze shifts because there is sufficient time for blinks to occur^7^,^8^. Furthermore, blinking is modulated depending on cognitive demands. Blinks are more frequent during times when attention is shifting, such as the end of a sentence^9^ or during a scene change^10^, and may facilitate attentional disengagement^11^. Blinking decreases during reading^12^ and when viewing salient scenes^13^. There is a reduced chance of missing critical visual information if blinks are inhibited during such cognitively-demanding tasks^7^.

Our growing understanding of blinking and its impact on visual and cognitive processes primarily comes from studies on human and nonhuman primates^2^. The factors influencing blinking in other species have not been rigorously investigated. Two studies have examined blinking in birds and they compared the blink rates of different species^14^,^15^ but did not systematically examine the blinks further. A third study found that American crows blinked less in response to possible danger^16^.

This study therefore investigated blinking in an avian species to examine the relationship between blinks, gaze shifts and context. Our limited understanding of blinking in birds partly stems from the challenge of collecting precise data on blinks and visual behavior in freely-behaving birds. To overcome this limitation, this study employed a novel and rigorous method of collecting such data by outfitting birds with a telemetric eye-tracker. In particular, blinking was studied in freely-moving peacocks (*Pavo cristatus*) that were wearing a telemetric eye-tracker that recorded their blinks and eye movements^17^. Peacocks are native to the Indian subcontinent and are subject to predation by terrestrial and aerial predators^18^,^19^,^20^. Because cognitively-demanding contexts can impact blinking behavior^7^, their blinks were compared in different contexts. Their blinks and eye movements before and after they were exposed to a taxidermy predator were compared. The relationship between their blinks and gaze shifts was examined with respect to predator presence.

## Results

**Figure 1:**
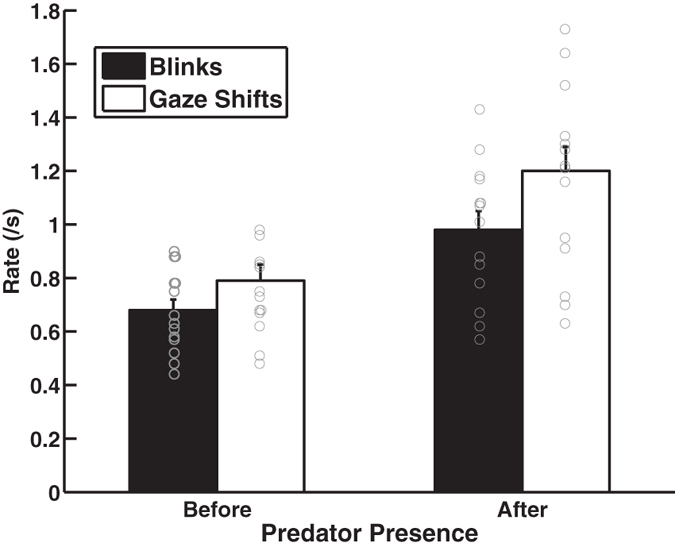
Blink and gaze shift rate before and after the predator was revealed.

**Figure 2:**
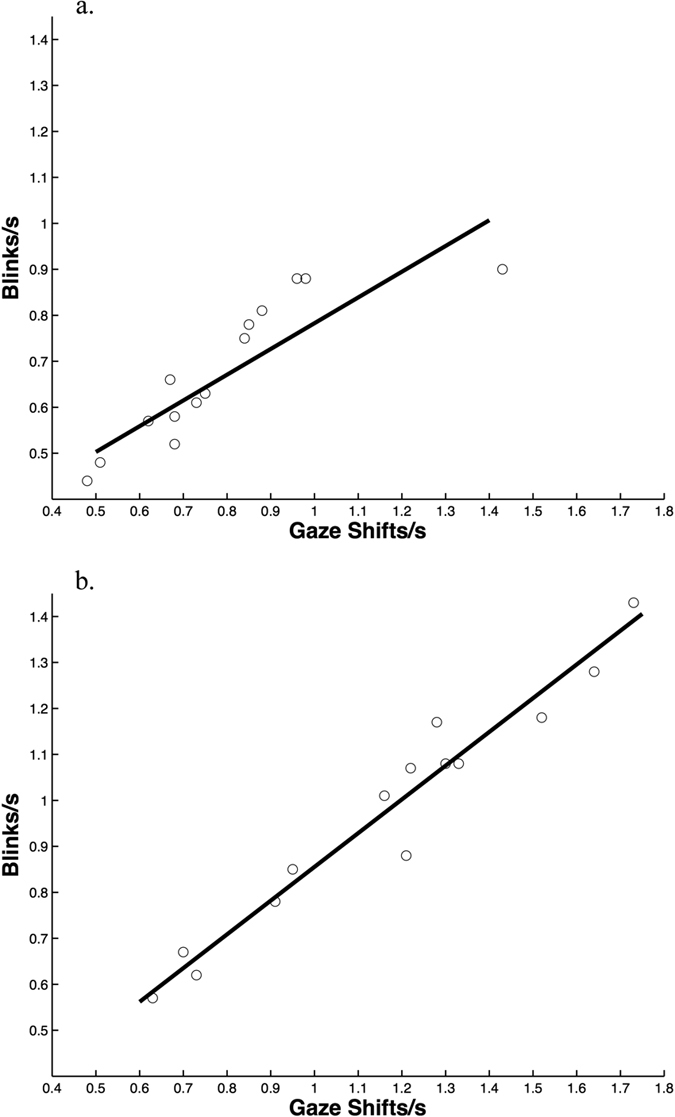
Blink and gaze shift rate were positively related (**a**) before the predator was revealed and (**b**) after the predator was revealed.

Blinks were short (0.11 ± 0.005 s) and started after a gaze shift began (0.02 ± 0.003 s after) and ended before a gaze shift finished (0.14 ± 0.008 s before). They were shorter after the predator was revealed compared to before the predator was revealed (F_1,13_ = 31.08, p < 0.001; before: 0.13 ± 0.008 s; after: 0.08 ± 0.005 s) just as the gaze shifts were shorter after the predator was revealed compared to before (F_1,13_ = 8.08, p = 0.014; before: 0.29 ± 0.02 s; after: 0.24 ± 0.01 s). Peacocks blinked more (blinks/s) and exhibited more gaze shifts (gaze shifts/s) after the predator was exposed compared to beforehand (blinks: F_1,13_ = 13.2, p = 0.003; gaze shifts: F_1,13_ = 10.42, p = 0.006; Fig. 1) but temperature (blinks: F_1,11_ = 0.39, p = 0.55; gaze shifts: F_1,11_ = 0.16, p = 0.69) and wind speed (blinks: F_1,11_ = 0.26, p = 0.62; gaze shifts: F_1,11_ = 0.00, p = 0.96) did not influence these rates. In only three trials did birds exhibit a higher gaze shift rate before compared to after the predator was revealed. Blink and gaze shift rates were similar during the first and second minute after the predator was exposed (blinks: F_1,13_ = 1.56, p = 0.23; gaze shifts: F_1,13_ = 1.57, p = 0.23). Blink rate was positively associated with gaze shift rate (before predator: R^2^ = 74.7; F_1,13_ = 39.3, p < 0.0001; after predator: R^2^ = 95.0; F_1,13_ = 223.06, p < 0.0001; Fig. 2). In fact, peacocks blinked during most of their gaze shifts (before predator: 87.2% ± 2.4%; after predator: 84.9% ± 1.6; range: 63–99%) but blinks were never observed without a gaze shift.

**Figure 3:**
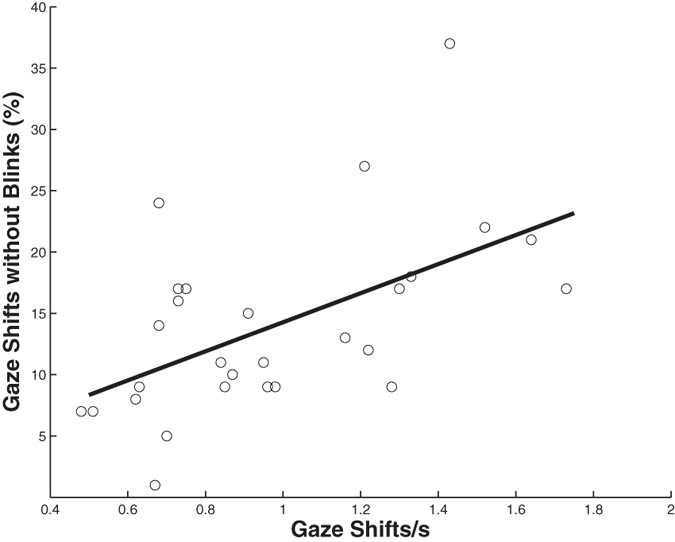
Peacocks were less likely to blink during a gaze shift when they exhibited a high gaze shift rate.

**Figure 4:**
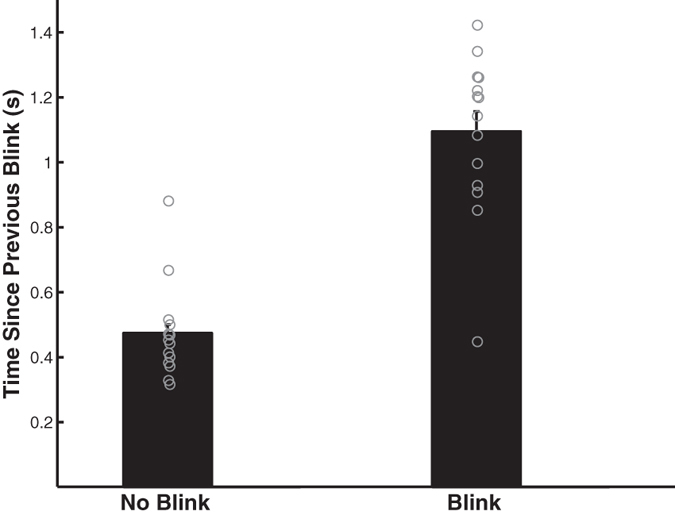
Peacocks were less likely to blink during a gaze shift when less time lapsed since their previous blink.

The percentage of gaze shifts that was not accompanied by blinks was unrelated to predator presence (F_1,11_ = 1.04, p = 0.33) and the interaction between predator presence and gaze shift rate (F_1,11_ = 2.20, p = 0.17). However, peacocks were less likely to blink during a gaze shift when the gaze shift rate was high (F_1,11_ = 15.42, p = 0.0024; Fig. 3). Similarly, blinks were less likely to occur when gaze shifts were of shorter duration (F_1,13_ = 13.95, p = 0.003). Peacocks were also less likely to blink during a gaze shift when less time lapsed between a gaze shift and their previous blink (F_1,13_ = 99.41, p < 0.0001; Fig. 4). They exhibited a shorter amount of time between their previous blink and a gaze shift after the predator was revealed compared to before the predator was revealed (F_1,13_ = 20.14, p = 0.0006) but the interaction between predator presence and blinks was unrelated to this time (F_1,13_ = 0.41, p = 0.54).

**Figure 5:**
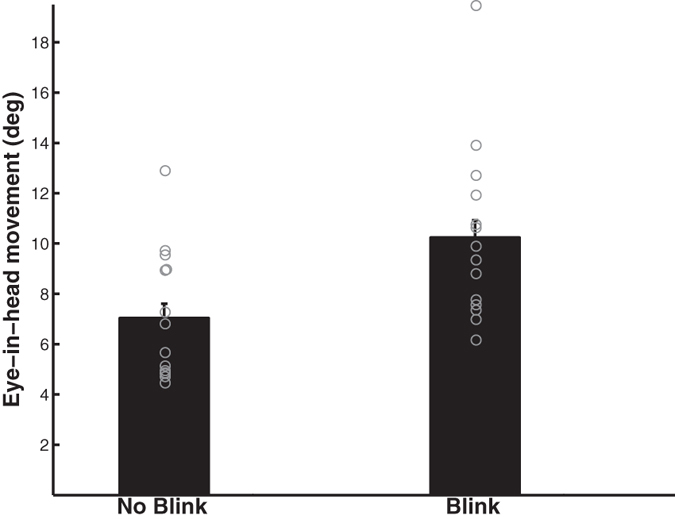
The size of eye-in-head movements was smaller when peacocks did not blink.

The size of their eye-in-head movements was smaller when they did not blink compared to when they did blink (F_1,13_ = 13.29, p = 0.003; Fig. 5). When the eye-in-head movement was greater than 15° (n = 42), 76% of gaze shifts were accompanied by blinks. The size of their eye-in-head movements was also smaller after the predator was revealed compared to before the predator was revealed (F_1,13_ = 5.08, p = 0.042). The interaction between predator presence and blinks was unrelated to eye-in-head movement size (F_1,13_ = 0.95, p = 0.35). The size of their head movements was unrelated to whether they blinked (F_1,13_ = 2.84, p = 0.12) but tended to be larger after the predator was revealed compared to beforehand (F_1,13_ = 3.9, p = 0.07). The interaction between predator presence and blinks was unrelated to head movement size (F_1,13_ = 0.20, p = 0.66).

**Figure 6:**
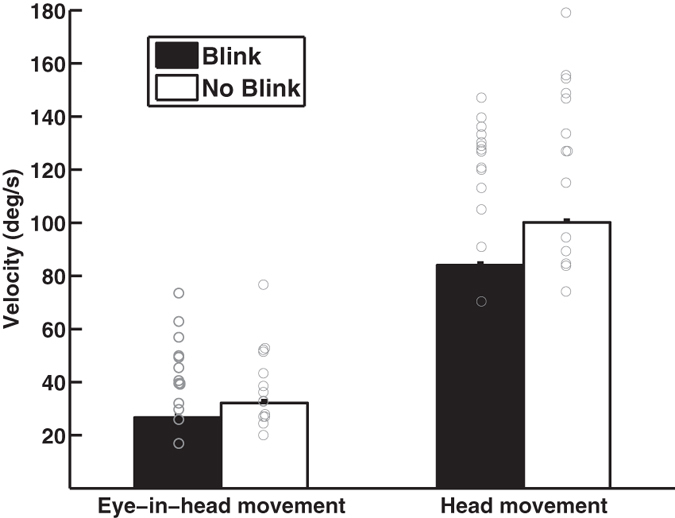
The velocity (lsmean) of eye-in-head and head movements was slower for gaze shifts with blinks compared to gaze shifts without blinks.

The velocity of the eye-in-head movement and head movement (amplitude of eye-in-head movement or head movement divided by gaze shift duration) was higher when the gaze shift was not accompanied by a blink (eye-in-head movement: F_1,13_ = 10.83, p = 0.006; head movement: F_1,13_ = 11.13, p = 0.0054; Fig. 6), after the predator was revealed compared to before (eye-in-head movement: F_1,13_ = 6.74, p = 0.022; head movement: F_1,13_ = 13.777, p = 0.0026), and when the amplitude of the gaze shift was large (eye-in-head movement: F_1,254_ = 1008.15, p < 0.0001; head movement: F_1,254_ = 1158.27, p < 0.0001). However, it was unrelated to the interaction between predator presence and blinks (eye-in-head movement: F_1,13_ = 0.40, p = 0.54; head movement: F_1,13_ = 0.54, p = 0.48). In 89% of these blinks, the nictitating membrane moved across the pupil but the eyelid did not. The remaining 11% of these blinks occurred before the predator was revealed and both the nictitating membrane and eyelid moved across the pupil (the eyelid never completely covered the pupil). Blink duration was shorter when only the nictitating membrane moved across the pupil compared to when both the nictitating membrane and eyelid moved across the pupil (nictitating membrane: 0.10 ± 0.004 s; nictitating membrane and eyelid: 0.20 ± 0.026 s; F_1,7_ = 31.55, p = 0.0008). Eye-in-head velocity did not differ depending on whether blinks were performed with the nictitating membrane versus the nictitating membrane and eyelid (nictitating membrane: 44.03 ± 3.26 deg s^−1^; nictitating membrane and eyelid: 34.48 ± 7.10 deg s^−1^; F_1,7_ = 2.02, p = 0.20) but head movement velocity was faster when only the nictitating membrane was involved (nictitating membrane: 128.34 ± 8.21 deg s^−1^; nictitating membrane and eyelid: 57.0 ± 12.7 deg s^−1^; F_1,7_ = 12.50, p = 0.0095).

Pupil size immediately before and after a gaze shift was similar regardless of predator presence (F_1,13_ = 3.01, p = 0.11) and the interaction between predator presence and blinks (F_1,13_ = 0.06, p = 0.81). Pupil size tended to be smaller after a gaze shift with a blink (F_1,13_ = 3.78, p = 0.07).

## Discussion

This is the first study to quantitatively examine the relationship between blinking, gaze shifts, and context in birds. Peacocks blinked during the majority of their gaze shifts but never blinked without a gaze shift. Furthermore, they inhibited their blinks the most when they were alert (high rate of gaze shifts). Their gaze shifts were shorter and faster in the presence of the predator but predator presence did not significantly impact blink inhibition.

Blink rates vary widely across species but have primarily only been studied in mammals. Some mammals exhibit low blink rates (e.g., 0.03 blinks/s in goats and 0.13 blinks/s in camels) whereas others exhibit higher rates (e.g., 0.27 blinks/s in elephants, 0.28 blinks/s in humans, and 0.37 blinks/s in cows; 12, 14). Blink rates are generally unrelated to light, humidity, heat and corneal anesthesia^21^. Similarly, blink rate in peacocks was unrelated to environmental factors (temperature and wind speed). Across primates, blink rates are higher in species with larger group sizes^2^. Blink rates in birds have rarely been investigated^14^,^15^,^16^. While similarities exist between avian and mammalian visual systems (e.g. ref. 22), which make comparisons between the two classes informative, there are also important differences. For example, birds exhibit smaller eye-in-head movements than primates^23^. Nevertheless, birds often exhibit higher blink rates than mammals. Diurnal birds tend to blink more often than nocturnal birds, with diurnal birds blinking an average of 0.20–0.89 blinks/s and nocturnal birds blinking 0.03–0.15 blinks/s^15^. Peafowl blink rates fall within the upper range of other diurnal birds, averaging 0.79 blinks/s during the period before the predator was revealed, but reaching higher rates, 1.17 blinks/s, after the predator was revealed. Turkeys (*Meleagris gallopavo*), a species closely related to peafowl, also exhibit high blink rates (0.72 blinks/s) that reach up to 0.85 blinks/s^15^. American crows blink less (0.49 blinks/s) while seeing a threatening person compared to a caring person (0.69 blinks/s;^16^).

Blinking does not occur randomly as it is strongly associated with saccades, rapid gaze shifts, in humans and other primates. During a blink, vision is impaired because the eyelids temporarily block visual information and activity in the visual cortex is suppressed^24^,^25^. During a saccade, visual information is likewise degraded as the eyes move between targets (saccadic suppression & motion blur;^26^,^27^). In humans, blinks are associated with 97% of saccadic gaze shifts that are larger than 33°^6^. In head-unrestrained monkeys, 65% of horizontal gaze shifts are associated with blinks^8^. Similarly, blinks in peacocks are strongly associated with gaze shifts. Over 80% of gaze shifts in peacocks were accompanied by a blink. Anecdotally, blinking in turkeys, white-winged doves and rock doves, is often associated with head movements^15^.

There are several hypotheses that could explain the positive association between blinks and gaze shifts. First, blinks occurring during gaze shifts, a time during which visual information is already limited, may minimize additional information loss. If animals blinked during intervals between gaze shifts they would not only have limited information during gaze shifts but also during blinks. Second, blinks and gaze shifts may be linked through general motor or neural linkage^28^. Based on clinical evidence and neurophysiological studies in humans, the neural circuitry underlying saccadic gaze shifts and blinks are linked^6^,^29^. Third, blinks may co-occur with gaze shifts to suppress blurred information. Extraneous visual information gathered during a gaze shift would be reduced if blinking is coincident with the gaze shift^30^. And fourth, blinks may be associated with gaze shifts to protect the eye against foreign debris or dry eyes^13^. Eyes may be exposed to more debris or become drier during gaze shifts compared to when they remain still. By blinking during times when exposure to debris is elevated and ocular surface humidity is low, blinks may help protect the eye^31^. Further experiments will be necessary to assess which hypothesis or hypotheses are driving the link between blinks and gaze shifts in peacocks.

The amplitude of the gaze shifts also influences the likelihood of blinks. In humans and monkeys, blinks are more likely to occur when gaze shifts are large^7^,^8^. Because larger gaze shifts require more time to complete than smaller gaze shifts, there is more time for a blink to occur without impairing vision^7^. In peacocks, blinks are also more likely to occur when gaze shifts are large.

While blinks are important for proper visual functioning^3^,^4^, they are associated with potential costs. While controlling for the size of the gaze shift, the average velocity of gaze shifts in peacocks is slower when a gaze shift occurs with a blink compared to when it occurs without a blink. A peacock’s blink decreased the eye-in-head movement velocity and head movement velocity by 17% and 16%, respectively. The velocity of eye-in-head movements did not differ relative to whether blinks involved both the nictitating membrane and eyelid or only the nictitating membrane but the velocity of head movements was faster when only the nictitating membrane was involved. Monkeys are also slower in making eye-in-head movement saccades when these saccades are accompanied by blinks. A blink slows down their saccades by 63%^32^. Rather than a blink slowing down the velocity of saccades, it is also possible that slower saccades elicit blinks.

Given that blinks are likely associated with costs, suppressing blinks could be beneficial under certain contexts. In primates, blinking varies depending on cognitive tasks, especially those involving attention and concentration^33^,^34^. Blinking tends to increase when memorizing sentences^35^, speaking^36^, and spending more time on a task^37^ but decrease when reading^12^ and engaging in difficult tasks^36^,^38^. Blinking may decrease as difficulty increases so that critical information is not missed^7^,^13^,^37^,^39^. Peacocks did not inhibit blinks any more often after the predator was exposed compared to before, which was unexpected given that the stationary predator was highly relevant and the birds would therefore likely want to monitor it closely. However, peacocks were less likely to blink during a gaze shift when the gaze shift rate was high, regardless of predator presence. Birds exhibit high rates of gaze shifts when they are alert^40^,^41^. Therefore, inhibition of blinks in peacocks was not specifically related to predator presence but was likely related to general vigilance levels.

High rates of gaze shifts allow birds to rapidly monitor different areas of their environment. Because birds do not exhibit smooth pursuit eye movements^42^, these gaze shifts enable them to monitor moving threats. They also enable birds to scan for threats positioned at scattered locations^40^. In addition, gaze shifts aid birds in object recognition^43^. Despite these benefits, high rates of gaze shifts likely increase visual disruption due to saccadic suppression and motion blur^26^,^27^. There are several hypotheses to explain why birds inhibit blinks during high rates of gaze shifts. First, they may inhibit blinks during high rates of gaze shifts to facilitate rapid gaze shifts. Because gaze shifts are faster when they are not accompanied by a blink, inhibiting blinks could allow animals to shift attention more quickly. Second, they may inhibit blinks during high rates of gaze shifts because it may not be necessary for the birds to moisten their eyes with each gaze shift when the gaze shifts occur at high rates. While blinks are important in moistening and protecting the corneal surface^3^,^4^, blinking at rates above a certain threshold may not provide additional benefits. Further experiments with multiple exemplars of the same or different type of predator that vary danger levels or cognitive demands would further increase our understanding of the role that blinking plays in avian vision. It would also be interesting to investigate whether blink rate in prey impacts predator behavior, with the prey potentially signaling to predators through blinking that the predators have been detected and should abort their attacks.

In humans, pupil size is also related to blinking. Pupil diameter increases after blinks regardless of whether they were accompanied by saccades^37^. This dilation may occur so that the brain can be prepared to acquire new information immediately following the blink^37^,^44^. In contrast, pupil diameter in peacocks did not vary before and after gaze shifts, suggesting that different mechanisms underlie pupil dilation in humans and birds. The difference in pupillary response could also be related to the fact that blinks in peacocks involve the nictitating membrane but this membrane does not exist in humans. Neural suppression during blinks and saccades^24^,^25^ may occur in peacocks such that they are unaware of these events, potentially explaining why their pupils did not change in size.

The mechanism that regulates blinking in birds is unexplored. In primates, the dopaminergic system plays an important role in blinking^45^. Dopamine is a neurotransmitter that impacts visual function, including contrast sensitivity, visual memory, visual-evoked potentials, and electroretinographic responses^46^. Monkeys and humans increase their blinking rate when administered dopamine agonists or medications that increase dopamine levels^45^,^47^. Experimental manipulation of dopamine levels in birds could uncover whether their blinking behavior is also under dopaminergic control.

## Materials and Methods

The blinks of 14 adult peacocks (males) were recorded using a telemetric eye-tracker (30 frames s^−1^) during a predator presentation experiment in Durham, North Carolina (36.01°N, 79.02°W; see 17 for further details about the eye-tracker and experimental design). For each trial, a peacock wearing the telemetric eye-tracker was transported to an outdoor testing cage (27 m perimeter) that contained another male and two females and he was allowed to freely move around the cage. After a minimum of 25 min, a taxidermy fox (*Vulpes vulpes*) was revealed for 5 min (see ref. 17 for further details on the experimental design). The eye-tracker recorded synchronized videos of one of the peacocks’ eye and the scene in front of that eye (the other eye had a patch over it). The videos were calibrated using an oculometric approach based on corneal reflections^34^. The calibrated videos consisted of the gaze of the bird overlaid on the scene camera. All experiments were conducted in accordance with the guidelines of Duke University’s Institutional Animal Care and Use Committee and were approved by Duke University (A169-11-07).

Blinking and gaze shifts were analyzed based on the video recordings from the eye-tracker during a 2-min period immediately before the predator was revealed and a 2-min period immediately after the predator was revealed. The total number of blinks and gaze shifts were manually counted during these periods and converted into rates. A blink was defined as when the eyelid or nictitating membrane moved across the pupil. A gaze shift was defined as when the eye and head rapidly changed positions (head movements were never observed without eye movements and vice versa; see also 23).

In addition, for both the before and after periods, five gaze shifts that were associated with a blink and five gaze shifts that were not associated with a blink were randomly selected from each trial (if fewer than 5 gaze shifts existed for a given trial, then all available gaze shifts were analyzed). Based on these selected gaze shifts, the duration of blinks, size of eye-in-head movements and head movements, and the latency between each gaze shift and the previous blink were calculated. The size of the eye-in-head movements was determined by calculating the angular distance that the calibrated gaze coordinate moved before and after the gaze shift; the size of the head movement was determined by calculating the angular distance that a stationary object within the scene camera moved before and after the gaze shift (see ref. 48 for further details on these calculations). Most of these gaze shifts, those that were and were not accompanied by blinks, involved shifts between environmental features (before predator: 78.1% (no blinks); 81.2% (with blinks); after predator: 82.6% (no blinks); 81.4% (with blinks); the other gaze shifts involved shifts between conspecifics, conspecifics and the environment, the focal peacock’s body/feathers and the environment, or different areas on the focal peacock’s body/feathers. Lastly, pupil size before and after a gaze shift were compared. Pupil size was standardized by multiplying the pupil diameter (pixels) from the eye camera by the width of the peacock’s eye (approximately 10 mm) and dividing by the number of pixels the width of the peacock’s eye occupied in the eye camera. Temperature (Celsius) and wind speed (mps) were obtained from a nearby weather station (35.97°N, 79.09°W; State Climate Office of North Carolina). Linear mixed models with repeated measures (SAS, Cary, NC, USA) were performed to assess the relationships between blinking, gaze shifts, and predator presence. Variables that did not meet normality assumptions were transformed.

## Additional Information

**How to cite this article**: Yorzinski, J. L. Eye blinking in an avian species is associated with gaze shifts. *Sci. Rep.* **6**, 32471; doi: 10.1038/srep32471 (2016).
